# Supplementary material for: Associations between Serum Kallistatin Levels and Markers of Glucose Homeostasis, Inflammation, and Lipoprotein Metabolism in Patients with Type 2 Diabetes and Nondiabetic Obesity
Source: Int J Mol Sci. 2024 Jun 6;25(11):6264. doi: 10.3390/ijms25116264 (PMC11173135; doi:10.3390/ijms25116264)
Supplement: Supplementary file 1 [file ijms-25-06264-s001.zip › Supplementary Figure S1.pdf]

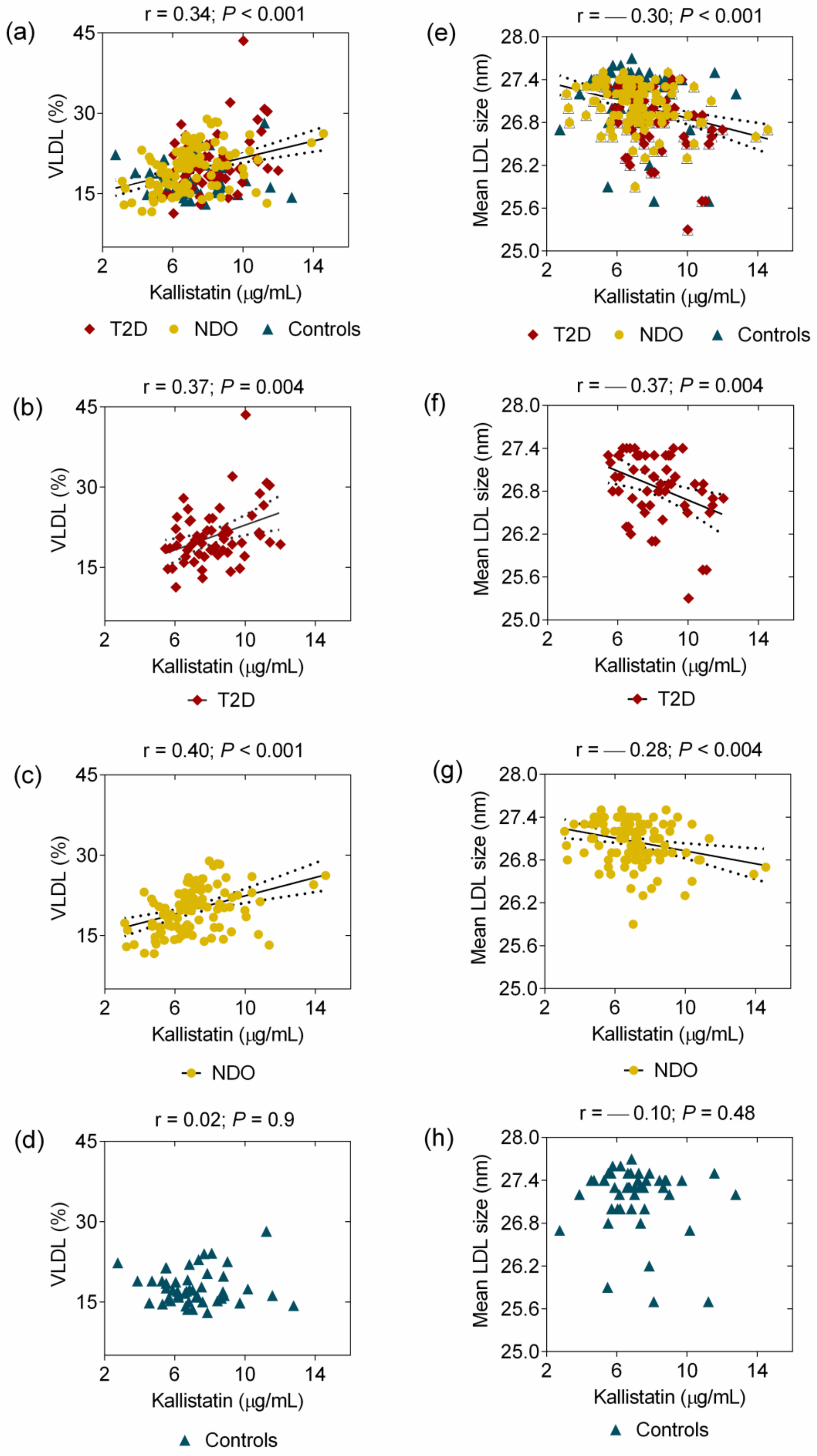
r = 0.34; P < 0.001
(a)
VLDL (%)
Kallistatin (μg/mL)
T2D
NDO
Controls
r = — 0.30; P < 0.001
(e)
Mean LDL size (nm)
Kallistatin (μg/mL)
T2D
NDO
Controls
r = 0.37; P = 0.004
(b)
VLDL (%)
Kallistatin (μg/mL)
T2D
r = — 0.37; P = 0.004
(f)
Mean LDL size (nm)
Kallistatin (μg/mL)
T2D
r = 0.40; P < 0.001
(c)
VLDL (%)
Kallistatin (μg/mL)
NDO
r = — 0.28; P < 0.004
(g)
Mean LDL size (nm)
Kallistatin (μg/mL)
NDO
r = 0.02; P = 0.9
(d)
VLDL (%)
Kallistatin (μg/mL)
Controls
r = — 0.10; P = 0.48
(h)
Mean LDL size (nm)
Kallistatin (μg/mL)
Controls

Supplementary Figure S1. Correlations of kallistatin with the percentage of very low-density lipoprotein (VLDL) subfractions (a) in overall; (b) in obese patients with type 2 diabetes (T2D; marked with red squares); (c) in nondiabetic obese (NDO; marked with yellow dots) and (d) in controls (marked with blue triangles). Correlations of kallistatin with mean low-density lipoprotein (LDL) size (e) in overall; (f) in obese patients with type 2 diabetes (T2D; marked with red squares); (g) in nondiabetic obese (NDO; marked with yellow dots) and (h) in controls (marked with blue triangles). Solid lines represent the linear regression bands and dotted lines represent the 95 confidence intervals.
