# Supplementary material for: Associations between Serum Kallistatin Levels and Markers of Glucose Homeostasis, Inflammation, and Lipoprotein Metabolism in Patients with Type 2 Diabetes and Nondiabetic Obesity
Source: Int J Mol Sci. 2024 Jun 6;25(11):6264. doi: 10.3390/ijms25116264 (PMC11173135; doi:10.3390/ijms25116264)
Supplement: Supplementary file 1 [file ijms-25-06264-s001.zip › Supplementary Figure S2.pdf]

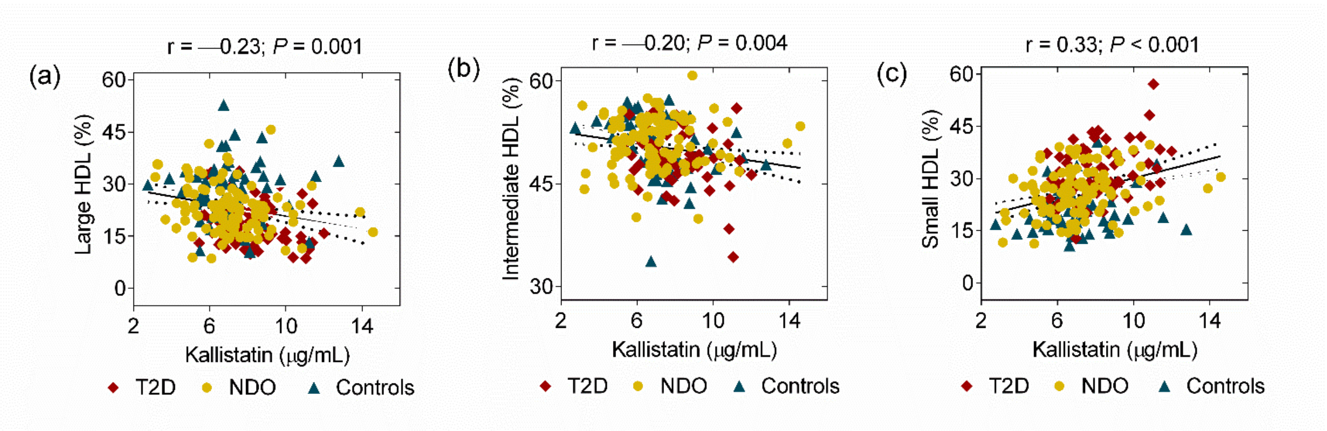

Supplementary Figure S2. Correlations of kallistatin with (a) the percentage of large high-density lipoprotein (HDL); (b) intermediate HDL and (c) small HDL subfractions in overall subjects. Red squares: obese patients with type 2 diabetes (T2D); yellow dots: nondiabetic obese (NDO) patients; blue triangles: controls. Solid lines represent the linear regression bands and dotted lines represent the 95 percent confidence intervals.
