# Supplementary material for: Associations between Serum Kallistatin Levels and Markers of Glucose Homeostasis, Inflammation, and Lipoprotein Metabolism in Patients with Type 2 Diabetes and Nondiabetic Obesity
Source: Int J Mol Sci. 2024 Jun 6;25(11):6264. doi: 10.3390/ijms25116264 (PMC11173135; doi:10.3390/ijms25116264)
Supplement: Supplementary file 1 [file ijms-25-06264-s001.zip › Table S1_kallistatin_RB.pdf]

Supplementary Table S1: Main medications of enrolled participants

	Controls (n=49)	NDO (n=106)	T2D (n=62)
Metformin (n, %)	0; 0	11; 10.4	45; 72.8
Insulin (n, %)	0; 0	0; 0	14; 35.5
GLP-1 RA (n, %)	0; 0	0; 0	14; 35.5
Statin (n, %)	0; 0	12; 11.3	26; 41.9
ACEI/ARB (n, %)	1; 2	41; 38.7	28; 45.2
CCB (n, %)	1; 2	15; 14.2	14; 22.6
Diuretics (n, %)	0; 0	22; 20.8	8; 12.9

Abbreviations: ACEI/ARB, angiotensin-converting enzyme inhibitors/angiotensin II receptor blockers; CCB, calcium channel blockers; GLP-1 RA, glucagon-like peptide-1 receptor agonists; NDO, nondiabetic obese patients; T2D, patients with type 2 diabetes mellitus
