# Supplementary figures and images for: Associations between Serum Kallistatin Levels and Markers of Glucose Homeostasis, Inflammation, and Lipoprotein Metabolism in Patients with Type 2 Diabetes and Nondiabetic Obesity
Source: Int J Mol Sci. 2024 Jun 6;25(11):6264. doi: 10.3390/ijms25116264 (PMC11173135; doi:10.3390/ijms25116264)

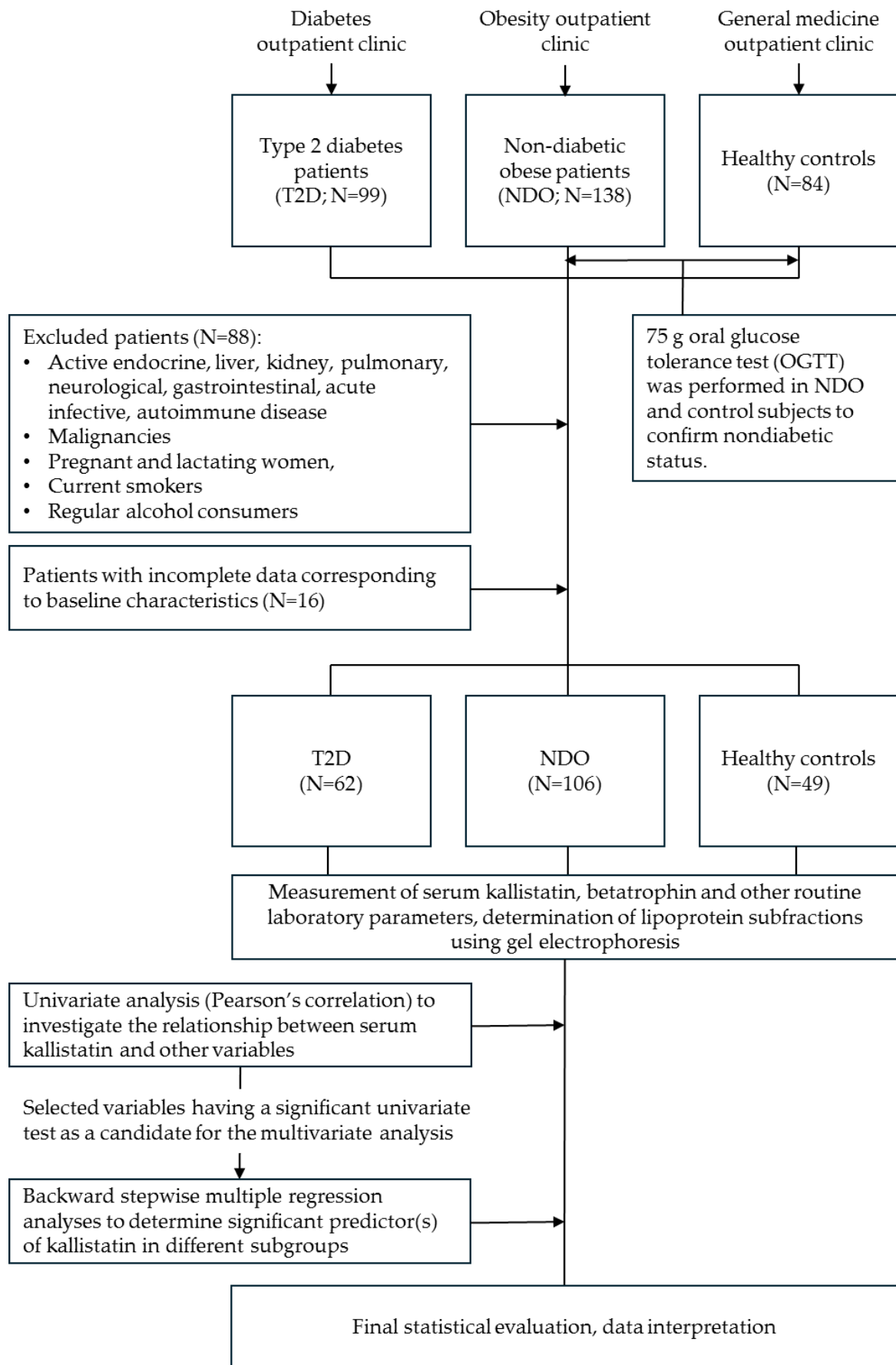

**Figure S3:** Study design flowchart of enrolled subjects

Supplement: Supplementary file 1 [file ijms-25-06264-s001.zip › Supplementary Figure S3.pdf]
